# Trunk Muscle Activation at the Initiation and Braking of Bilateral Shoulder Flexion Movements of Different Amplitudes

**DOI:** 10.1371/journal.pone.0141777

**Published:** 2015-11-12

**Authors:** M. Eriksson Crommert, K. Halvorsen, M. M. Ekblom

**Affiliations:** 1 Faculty of Medicine and Health, Örebro University, Örebro, Sweden; 2 The Swedish School of Sport and Health Sciences (GIH), Stockholm, Sweden; 3 School of Technology and Health, KTH Royal Institute of Technology, Stockholm, Sweden; 4 Department of Information Technology, Uppsala University, Uppsala, Sweden; 5 Department of Neuroscience, Karolinska Institutet, Stockholm, Sweden; Duke University, UNITED STATES

## Abstract

The aim of this study was to investigate if trunk muscle activation patterns during rapid bilateral shoulder flexions are affected by movement amplitude. Eleven healthy males performed shoulder flexion movements starting from a position with arms along sides (0°) to either 45°, 90° or 180°. EMG was measured bilaterally from transversus abdominis (TrA), obliquus internus (OI) with intra-muscular electrodes, and from rectus abdominis (RA), erector spinae (ES) and deltoideus with surface electrodes. 3D kinematics was recorded and inverse dynamics was used to calculate the reactive linear forces and torque about the shoulders and the linear and angular impulses. The sequencing of trunk muscle onsets at the initiation of arm movements was the same across movement amplitudes with ES as the first muscle activated, followed by TrA, RA and OI. All arm movements induced a flexion angular impulse about the shoulders during acceleration that was reversed during deceleration. Increased movement amplitude led to shortened onset latencies of the abdominal muscles and increased level of activation in TrA and ES. The activation magnitude of TrA was similar in acceleration and deceleration where the other muscles were specific to acceleration or deceleration. The findings show that arm movements need to be standardized when used as a method to evaluate trunk muscle activation patterns and that inclusion of the deceleration of the arms in the analysis allow the study of the relationship between trunk muscle activation and direction of perturbing torque during one and the same arm movement.

## Introduction

Rapid voluntary arm movements at the shoulder perturb postural equilibrium and challenge the stabilization of the spine via reactive forces and torques. As such, rapid shoulder flexions, either uni- or bilateral, have been frequently used to study leg and trunk muscle activation patterns (e.g. [1,2]). Special attention has been given to the anticipatory muscle activation supposedly aimed to counteract and minimize the impact of the disturbance from the arm movement [1–3]. It has been reported that in healthy persons, the first trunk muscle to be activated at the initiation of a rapid shoulder flexion is the innermost abdominal muscle, the transversus abdominis (TrA), and that this early activation was absent in a group of patients with low back problems [4]. Since then, the shoulder flexion task has been used extensively as an experimental model to describe trunk muscle activation patterns [5–9], to compare trunk muscle activation patterns between categories of people [4,10,11] and to investigate effects of interventions [12,13]. Even though, any experimental model should be robust and well standardized, the end position of the arm movement is often not controlled, with the motivation that it is the initiation of the movement that is under study. Yet, increased movement amplitude increases the duration of acceleration of the arm and results in higher velocity, thus a larger perturbation. This means that arm movements with different endpoints might induce perturbations of different magnitude to the trunk. In line with this, there is a report of increasing torque, about the ankles, with increasing asymmetrical (right arm is moved towards flexion and left arm simultaneously towards extension) arm movement amplitudes from 5 to 40 °, with associated increase in trunk muscle EMG amplitude [14]. Furthermore, it has been shown that keeping the arm movement amplitude the same but decreasing the speed of a unilateral shoulder flexion from maximal to submaximal, delays TrA activation [15]. However, detailed mechanical description of symmetrical (bilateral) shoulder flexion movements with maximal speed but of different amplitudes, and the impact on trunk muscle activation patterns, such as onset and amplitude of activation, is still lacking.

A kinetic analysis has demonstrated a ventrally directed torque on the trunk at the level of the shoulders at the initiation of rapid shoulder flexion and a dorsally oriented torque at the initiation of rapid shoulder extension [16]. Since the type of muscle activity needed to decelerate a shoulder flexion is similar to that required producing a shoulder extension, the direction of the reactive torque in the two situations should also be similar, i.e. dorsally oriented. However, this has not been investigated previously, but has implications for choosing the intervals for e.g. muscle activation amplitude measurements during a shoulder flexion. Also, differences between acceleration and deceleration of arms may vary with movement amplitude, depending on e.g. varying degree of assistance from gravity during deceleration depending on arm position. It has previously been shown that the activation of the superficial trunk muscles at the initiation of rapid arm movements is direction specific, creating a torque with the opposite direction from the reactive torques resulting from the limb movements [1,2,17]. Thus, these muscles should be active in a phasic manner during the completion of the arm movement. In contrast, TrA has shown an activation pattern that is independent on the direction of the torque in the sagittal plane, both regarding onset latency in advance of rapid shoulder flexion or extension movements [18] and activation amplitude in static flexed or extended arm positions [19]. It is yet to be shown if this direction independency is present also when comparing the acceleration and deceleration phase within a rapid shoulder flexion movement.

The purpose of the present study was to describe the activation patterns, both timing and amplitude, of four trunk muscles during the acceleration and deceleration of rapid bilateral shoulder flexion movements of different amplitudes. Further, using inverse dynamics analysis, the aim was to relate the muscle activation to the magnitude and direction of the perturbation to the trunk at shoulder height caused by the various arm movements. The main hypotheses were that the onset latency as well as the amplitude of trunk muscle activation would be dependent on the amplitude of the arm movement and the associated magnitude of trunk perturbation, and that the activation amplitude of TrA would be independent of the phase (acceleration/deceleration) of the arm movement.

## Materials and Methods

### Subjects

Eleven healthy male volunteers (mean ±1 SD age 28 ± 4 years, height 1.81 ± 0.08 m, mass 80 ± 8.4 kg) participated. Subjects were recruited via advertisements on campus and consisted of staff and students at the Swedish School of Sport and Health Sciences. The subjects had not experienced any low back pain in the last 3 years, where low back pain was defined as having to stay home from work or school, or avoid participating in normal activities due to pain from the back. Furthermore, the subjects had no muscular-, skeletal-, neurological- or inflammatory disease and had not had any surgery performed on the trunk. All subjects read and signed a written consent form prior to participation. The protocol was approved by the Regional Ethical Review Board in Stockholm (Reg. no. 2009/438-31) and the procedures performed in accordance to the 1964 declaration of Helsinki.

### Experimental procedure

**Fig 1:**
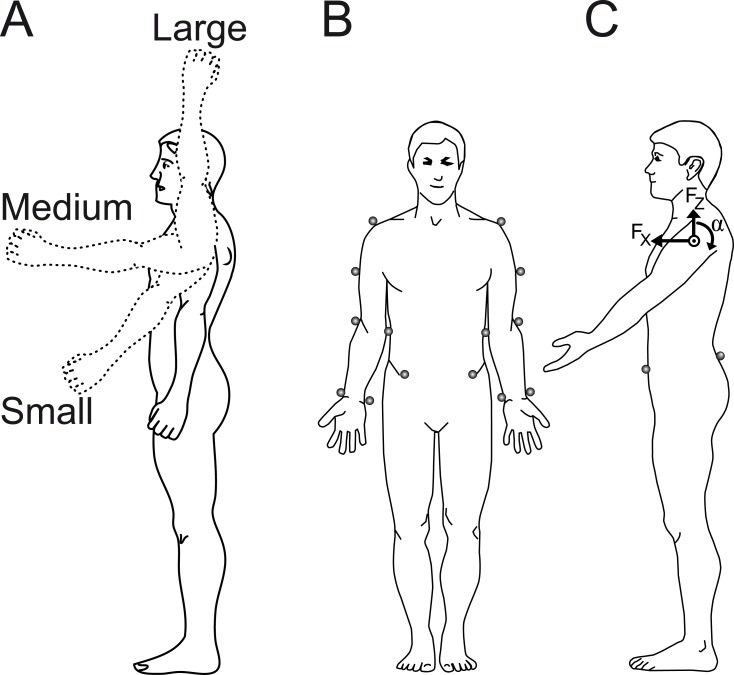
The experimental set-up. In (A) the three different arm movement amplitudes are illustrated with the solid arm showing the start position of all movements and the three dashed arms representing the three different end positions. In (B) and (C), the marker positions used are shown and in (C) also the reactive torque (α) on the trunk around the centre of rotation and the linear forces in the antereo-posterior (F_x_) and caudo-cranial (F_z_) directions during the deceleration of an arm movement is illustrated.

The subjects stood barefoot on the floor with arms along the sides and were instructed to raise both arms as fast as possible to one of three target end positions; 45°, 90° and 180° in relation to the vertical (Fig 1) in response to a beep signal. Each position was targeted three times consecutively with the order of end positions randomized between subjects. Each subject was instructed to keep his eyes on a picture of the current end position placed at eye level in front of him. After approximately 5 s, the subject received a verbal command and lowered his arms back to the start position. Two practice movements were performed to ensure that the movement was performed according to instructions. Given the denotations in Fig 1, the total torque and linear forces on the trunk at shoulder height caused by the arm movement was calculated as M = α_left_ + α_right_, Fx = Fx_left_ + Fx_right_ and Fz = Fz_left_ + Fz_right_. The effects of gravity are included in α.

Last in the experimental protocol, the subjects performed 3 static maximum voluntary contractions in upright standing, front or back to a wall: attempted trunk flexion, Valsalva manæuvre (maximal voluntary pressurization of the abdomen) with a superimposed attempted trunk flexion, and attempted trunk extension. All maximum voluntary contractions lasted approximately 5 s and were performed twice with the resistance provided by a broad belt placed around the chest at armpit height and firmly fixed to the wall. The efforts were performed without visual feedback but with verbal encouragement by the investigators.

### Electromyographic recordings

Bilateral intra-muscular EMG signals were recorded with fine-wire electrodes placed in TrA, insertion point in the mid-axillary line, 2 cm caudal to the twelfth rib, and obliquus internus (OI), insertion point 2 cm ventral of the mid-axillary line and 2 cm caudal to the twelfth rib. The electrodes were made of Teflon coated seven-stranded silver wire (0.4 mm in diameter, Leico Industries, USA) with 2 mm of the coating removed at the tip. Each wire was inserted using sterilized needles (0.70 x 88 mm for TrA and 0.60 x 60 mm for OI) under ultrasound guidance (GE Logic 9, Transducer 12 MHz, UK). The two hooked electrode tips within each muscle were placed with an inter-electrode distance of about 5 mm. Needles were inserted obliquely from the side to minimize the risk that movement within or between muscles would displace the electrodes. After electrode placement, the needles were gently removed. Bilateral surface EMG recordings (self-adhesive Ag/AgCl electrodes (Blue Sensor, Ambu, Denmark, interelectrode distance 2 cm) were made from rectus abdominis (RA), 2 cm lateral to the umbilicus, erector spinae (ES), 3 cm lateral to the L3 spinous process and from the anterior deltoids. A ground electrode was placed over the spinous process of C7. Besides the adhesive, tape was used to fixate all surface electrodes to the skin. All EMG signals were amplified 1000 times (Myosystem 2000, Noraxon, USA, for TrA, IO, ES and RA, and NL 824, Digitimer Ltd, UK, for deltoids) and band-pass filtered between 10 and 1000 Hz with a 1^st^ order Bessel filter (NL 125, Digitimer Ltd, UK) with a notch filter at 50 Hz.

The EMG signals were sampled in parallel on two systems. For the latency analysis, separate collection software was used with a sampling rate of 2 kHz (Spike2, version 5.15, Cambridge Electronic Design, UK) and for the EMG amplitude measurements, the signals were collected on the kinematic recording system at 1.5 kHz (Qualisys Track Manager, Qualisys AB, Sweden). A power spectrum analysis of one of the files sampled at 2 kHz revealed very little power in the signals above 0.75 kHz. This indicates that the sampling frequency of 1.5 kHz of the kinematic recording system should be roughly in agreement with the Nyquist theorem of a sampling frequency of twice the frequency of the signal, and the risk of aliasing should be minimal.

### Kinematic recordings

3D kinematic recordings were made with an optoelectronic system (Pro reflex, MCU 1000, Qualisys AB, Sweden) and collected with computer software (Qualisys Track Manager, Qualisys AB, Sweden). The set-up involved 7 cameras and 16 reflexive markers (for marker locations see Fig 1B and 1C). The markers had a diameter of 19 mm and during calibration the residuals of each camera were below 2 mm. Kinematic data were sampled at 150 Hz and low pass filtered at 20 Hz. The anatomical location of the markers will be given in the text ahead under the subheading Kinematics and Kinetics.

### Data analysis

#### EMG onsets

EMG onsets were defined as the instant when the rectified EMG signal rose above a threshold level set to the average baseline signal plus 1.4 SD, calculated during a period of 80 ms prior to the trigger signal (beep), and stayed above this level for at least 25 ms [20]. The signal was allowed to fall beneath the threshold level for no more than 3 ms within the 25 ms without affecting the onset definition [21]. For left and right deltoids, the search for onsets started at the trigger signal; for the rest of the muscles, the search started 100 ms prior to the onset of the ipsilateral deltoid and lasted for 200 ms. The investigator was blinded as to which muscle was displayed while performing this analysis. EMG onsets were first determined automatically using a custom made algorithm in Spike2 software and then checked manually [22]. For all movement amplitudes, the mean onset of the three trials for each muscle, relative the onset of the ipsilateral deltoid, was used in the statistical analysis.

#### EMG amplitudes

**Fig 2:**
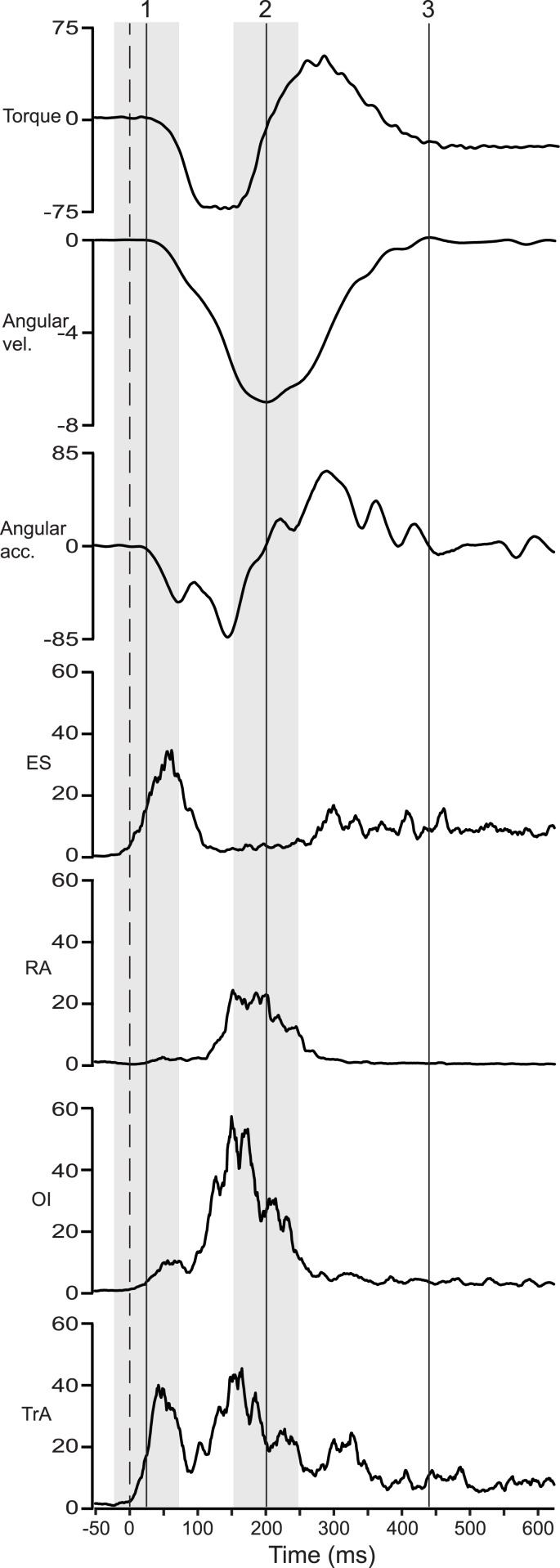
Recordings from one subject. Mean angular velocity and acceleration of shoulder flexion, mean reactive torque on the trunk at shoulder height, and mean normalized EMG in % of that at maximum voluntary contraction of the four different trunk muscles for three repetitions of a medium sized arm movement. The dashed line indicates mean onset of deltoid EMG and the shaded areas illustrate the two intervals of EMG amplitude measurements. The solid vertical lines indicate: 1 = start of acceleration of shoulder flexion, 2 = start of deceleration of shoulder flexion and 3 = end of shoulder flexion. Note the two peaks of activation in TrA corresponding to both acceleration and deceleration of the arm movement.

EMG amplitudes were calculated as root mean squares in two 100 ms intervals, one starting 50 ms prior to the start of the bilateral arm acceleration and the other 50 ms before the start of the bilateral arm deceleration (see definitions of events below and illustrations in Fig 2). The amplitudes were normalized to the highest EMG root mean square value, obtained with a 1 s sliding window for each muscle during the maximum voluntary contractions. For all movement amplitudes, the mean EMG amplitude of the three trials was measured for each muscle at the start of acceleration and at the start of deceleration and used in the statistical analysis.

#### Kinematics and kinetics

The reflexive markers were labelled to a large extent by an automated process with subsequent manual labelling. Kinematic analysis and kinetic calculations were performed using custom written code (Matlab, The Mathworks Inc, Natick, MA, USA) and commercial computer software (Visual3D, C-motion, Germantown, MD, USA). Two segments defined each arm. The markers on the processus styloideus radii, processus styloideus ulnae, and the medial and lateral epicondyles of the humerus defined the forearm. The markers on the medial and lateral epicondyles of the humerus, lateral upper arm and acromion defined the upper arm. The markers on the left and right acromion and the left and right anterior superior iliac spine defined the trunk. The joint centre for the whole arm was taken as the centre of rotation at the shoulder. The masses of the individual segments were defined in proportions of the total body mass according to Dempster [23]. The inertial properties of the segments, including their centres of mass, were defined from the segment model (default settings in Visual3D) assuming a homogenous distribution of the mass of each segment. Truncated cones represented the shape of the segments.

After defining the local coordinate systems, joint torques were calculated using inverse dynamics, by which measured segment positions, velocities and accelerations (from marker data) are combined with the model of the body segments (articulations and inertial properties) to compute the joint torques and linear forces necessary to produce the observed movement [24]. Only the linear forces in the antereo-posterior direction (Fx) and caudo-cranial direction (Fz) together with the torques in the sagittal plane was considered in this study. The medio-lateral forces were excluded since we assumed that the forces in this direction, to a large degree, would cancel each other out due to the symmetry of the movement. The total torque, Fx and Fz that perturb the trunk at shoulder level due to the movements of the arms was calculated by adding the two sides together.

Three events describing each unilateral arm movement were defined relative the angular acceleration at each shoulder (Fig 2). Firstly, start of arm acceleration was defined as the instant when the angular acceleration at the shoulder (upper arm relative the trunk) rose above a threshold level of a baseline average plus 2 SD and stayed above this level for 20 ms. Secondly, start of deceleration of the arm movement was defined as the instant when the angular acceleration of the shoulder crossed the 0 line. During the medium (example in Fig 2) and large amplitude movements, the signal often oscillated around 0 (constant velocity) for a period of time. When this occurred, the last crossing was defined as the start of deceleration. Thirdly, end of movement was defined as the instant when the angular acceleration returned to 0. Also at the end of movement the signal could oscillate around 0. The crossing that correlated best with the instant when the concomitant angular velocity signal of the shoulder movement returned to 0 was then used. The movement events were first defined automatically with a computer and then checked manually.

The start of the bilateral arm movement was defined as the start of acceleration of the first arm to move, the onset of deceleration as the mean for the start of deceleration between the left and right arm, and the end of movement as the last arm to reach the defined end position. The mean torque, Fx and Fz during the acceleration phase (between start of acceleration and start of deceleration) and the deceleration phase (between start of deceleration and the end of movement) were calculated. The mean torque and the linear forces Fx and Fz during the acceleration and deceleration phase, respectively, was multiplied with the duration of each phase to obtain the angular and linear impulses (Fx-impulse and Fz-impulse) imposed on the trunk by the arm movement. For each of the phases and movement amplitudes, the mean angular and linear impulses of the three trials were calculated and used in the statistical analysis as a measure of the magnitude of the perturbation.

### Statistical analysis

Statistical analyses were performed using PASW Statistics 18.0 (SPSS Inc., USA). Regarding EMG latency, a three-way Mixed model ANOVA was used with latency as the dependent variable and muscle, side and movement amplitude as within-subject factors. The EMG amplitude was analyzed with a four-way Mixed model ANOVA with normalized EMG amplitude as the dependent variable and muscle, side, movement amplitude and phase (acceleration/deceleration) as within-subject factors. The residuals were positively skewed when histograms and normal plots were visually checked. Thus, log transformation and square root transformations were performed. The square root transformation was deemed the most appropriate and used in the analysis of the EMG amplitude data. For the kinematic and kinetic variables, several two-way Repeated measures ANOVAs were used with peak and mean angular acceleration/deceleration, mean angular velocity, duration, mean torque, linear forces (Fx and Fz), angular impulse and linear impulses (Fx-impulse and Fz-impulse) as the dependent variables and movement amplitude and phase as within subject factors. Where a main effect or interaction was found in the main ANOVAs, subsequent post hoc pair-wise comparisons were performed with Bonferroni corrections. Possible differences regarding timing of acceleration/deceleration and arm movement amplitudes between the left and right arm were tested with one-way ANOVAs. The level of significance was set to p<0.05 for all statistical analyses.

## Results

No main effects were seen for side in the EMG latency and amplitude analyses. Therefore, the results are reported as mean values of the left and right side.

### General movement characteristics

All kinematic and kinetic variables are shown in Table 1. Regarding the end positions of the arm movements, the deviations from the target angles were less than ± 6° for all movement amplitudes, and there was no consistent difference between the left and right arm. The timing differences between the left and right arm ranged 0–2 ms for the start of acceleration, 6–16 ms for the start of deceleration and 5–10 ms for the end of the arm movement, respectively, and they were not statistically significant for any event. The peak velocity and the total duration of the movements increased with arm movement amplitude and averaged 281 (50), 487 (96), and 596 (73) °^/^s, and 0.39 (0.06), 0.45 (0.07) and 0.56 (0.06) s, respectively.

**Table 1:** Kinematic and kinetic variables (means and SD) during the acceleration and deceleration phases of the three bilateral arm movement amplitudes.

	Acceleration			Deceleration		
	Small	Medium	Large	Small	Medium	Large
Peak acc/dec_ang_ (°/s^2^)	5028 (2563) ^b^	5959 (1507) ^c^	7367 (1923) ^b^ ^c^ *	4685 (1662) ^a^	5791 (1277) ^a^	5912 (1576) *
Mean acc/dec_ang_ (°/s^2^)	1634 (451) ^a^ *	2297 (480) ^a^ *	2047 (565) *	1401 (497) ^a^ ^b^ *	1829 (471) ^a^ *	2115 (499) ^b^ *
Mean vel_ang_ (°/s)	112.2 (12.0) ^a^ ^b^ *	205.2 (25.0) ^a^ ^c^ *	331.1 (29.6) ^b^ ^c^ *	88.0 (15.8) ^a^ ^b^ *	164.4 (22.1) ^a^ ^c^ *	220.9 (46.5) ^b^ ^c^ *
Duration (s)	0.17 (0.02) ^a^ ^b^ *	0.19 (0.02) ^a^ ^c^ *	0.28 (0.04) ^b^ ^c^	0.22 (0.04) ^b^ *	0.25 (0.06) *	0.28 (0.05) ^b^
Mean torque (Nm)	-29.2 (10.1) ^a^ ^b^ *	-43.0 (11.8) ^a^ *	-40.4 (7.8) ^b^ *	12.4 (8.7) ^b^ *	14.5 (8.7) ^c^ *	25.5 (8.1) ^b^ ^c^ *
Linear force X (N)	28.9 (9.8) ^b^ *	34.0 (11.4) ^c^ *	-5.1 (8.6) ^b^ ^c^ *	-25.1 (10.8) ^b^ *	-28.1 (9.0) ^c^ *	7.0 (12.2) ^b^ ^c^ *
Linear force Z (N)	18.1 (10.5) ^a^ ^b^ *	36.7 (8.6) ^a^ *	46.0 (10.6) ^b^ *	-10.8 (11.3) ^a^ ^b^ *	-30.8 (9.0) ^a^ ^c^ *	-47.3 (9.6) ^b^ ^c^ *
Impulse_ang_ (Nms)	-4.8 (1.3) ^a^ ^b^ *	-8.2 (1.7) ^a^ ^c^ *	-11.0(2.0) ^b^ ^c^ *	2.3 (1.4) ^b^ *	3.2 (2.0) ^c^ *	6.7 (1.5) ^b^ ^c^ *
Impulse_Fx_ (Ns)	4.8 (1.3) ^a^ ^b^ *	6.3 (1.8) ^a^ ^c^ *	-1.7 (2.6) ^b^ ^c^ *	-5.1 (1.5) ^a^ ^b^ *	-6.7 (2.0) ^a^ ^c^ *	1.3 (2.7) ^b^ ^c^ *
Impulse_Fz_ (Ns)	3.0 (1.5) ^a^ ^b^ *	7.0 (1.6) ^a^ ^c^ *	12.4 (2.0) ^b^ ^c^ *	-2.3 (2.0) ^a^ ^b^ *	-7.3 (1.9) ^a^ ^c^ *	-12.8 (1.9) ^b^ ^c^ *

Negative values indicate flexion direction for the torque, anterior direction for Fx, and upward direction for Fz.

^a^ = significant difference between small and medium amplitude movements within phase.

^b^ = significant difference between small and large amplitude movements within phase.

^c^ = significant difference between medium and large amplitude movements within phase.

* = significant difference between acceleration and deceleration phases within movement amplitude.

### Acceleration phase

A flexion angular impulse on the trunk at shoulder height was observed during the acceleration phase for all arm movement amplitudes, with the angular impulses becoming significantly larger in magnitude with increasing arm movement amplitude, ranging 4.8 (1.2)– 11.0 (2.0) Nms (Table 1). During the two smaller arm movement amplitudes the linear Fx-impulse was oriented posteriorly and increased with arm movement amplitude. The net direction changed to anterior during the large arm movement amplitude (Table 1). The linear Fz-impulse was oriented downwardly and increased in magnitude with increased arm movement amplitude, ranging 3.0 (1.5)– 12.4 (2.0) Ns (Table 1).

**Fig 3:**
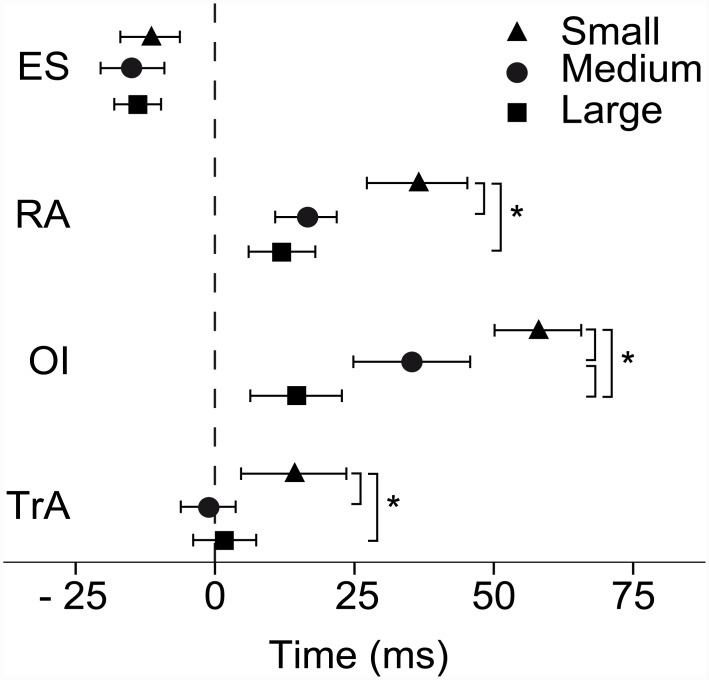
Bilateral mean onset latencies in ms (95% CI) for the four trunk muscles at the initiation of bilateral shoulder flexion for the three movement amplitudes. The dashed line indicates the mean onset of left and right deltoid. * Indicates a significant difference within muscles between arm movement amplitudes

For EMG latency in relation to onset of deltoid activation, there was a significant interaction between muscle and movement amplitude (Fig 3). All abdominal muscles responded significantly faster at the large compared to the small arm movement amplitude. Onset latencies for TrA, OI and RA being 14 (37), 58 (31) and 36 (33) ms in the small; -1 (19), 35 (36) and 16 (21) ms in the medium; and 2 (23), 15 (30) and 12 (23) ms in the large amplitude movement (Fig 3). In addition, OI displayed shorter onset latency at the large compared to the medium amplitude movement. ES did not vary in timing of activation between movement amplitudes with onset latencies of -12 ms (22), -15 ms (23) and -14 ms (17) for the small, medium and large movement amplitude respectively.

Regarding activation magnitude, there was a significant interaction between muscle, phase and movement amplitude. At the start of arm acceleration, the magnitude of muscle activation varied with the arm movement amplitude for TrA and ES with greater activation at the large compared to the small amplitude movements (Table 2). For TrA, there was also a difference with higher activation at the medium compared to the small movement amplitude (Table 2). No differences in muscle activation levels between movement amplitudes were present for OI or RA.

**Table 2:** Mean normalized EMG amplitude as % of that at maximum voluntary contraction (SD) for each muscle and arm movement amplitude, at the start of arm acceleration and deceleration, respectively.

	Acceleration			Deceleration		
	Small	Medium	Large	Small	Medium	Large
ES	10 (4) ^b^	16 (5) *	22 (7) ^b^ *	9 (4) ^b^	6 (3) *	4 (2) ^b^ *
TrA	17 (13) ^a^ ^b^	26 (20) ^a^	34 (26) ^b^	20 (14) ^b^	26 (22)	30 (21) ^b^
OI	6 (5) *	7 (7) *	9 (10) *	28 (16) *	34 (13) *	27 (19) *
RA	3 (2) *	3 (2) *	3 (2) *	11 (6) *	14 (8) *	13 (9) *

^a^ = significant difference between small and medium amplitude movements within muscle and phase.

^b^ = significant difference between small and large amplitude movements within muscle and phase.

* = significant difference between start of acceleration and start of deceleration within muscle and movement amplitude.

### Deceleration phase

During the deceleration phase, the angular impulse to the trunk at shoulder height was reversed to extension. The angular impulse during the deceleration of the large arm movement was significantly greater than during the small and medium arm movements, with the angular impulse magnitude ranging, 2.3 (1.4)– 6.7 (1.5) Nms (Table 1). The linear Fx-impulse was oriented anteriorly and increased in magnitude with arm movement amplitude during the small and medium arm movement. During the large movement amplitude the net direction changed towards posterior (Table 1). The linear Fz-impulse was oriented upwards across all movement amplitudes and increased in magnitude as arm movement amplitude increased, ranging -2.3 (2.0)–-12.8 (1.9) Ns (Table 1).

At the start of deceleration of the arm movement, TrA showed higher activation levels at the large movement compared to the small, whereas ES displayed the opposite with less activation in the large movement compared to the small (Table 2). No difference in activation of OI or RA was evident between movement amplitudes.

### Acceleration vs. deceleration

Comparing the magnitude of muscle activation at the start of arm acceleration to that at the start of deceleration it was shown that TrA was activated to the same magnitude at both events, i.e. regardless flexion or extension impulse to the trunk at shoulder height (Table 2). OI and RA showed higher activation levels at the start of the deceleration phase compared to the start of the acceleration phase across arm movement amplitudes. ES displayed the opposite, with higher activation at the start of the acceleration phase compared to the deceleration phase, except at the small arm movement amplitude.

**Fig 4:**
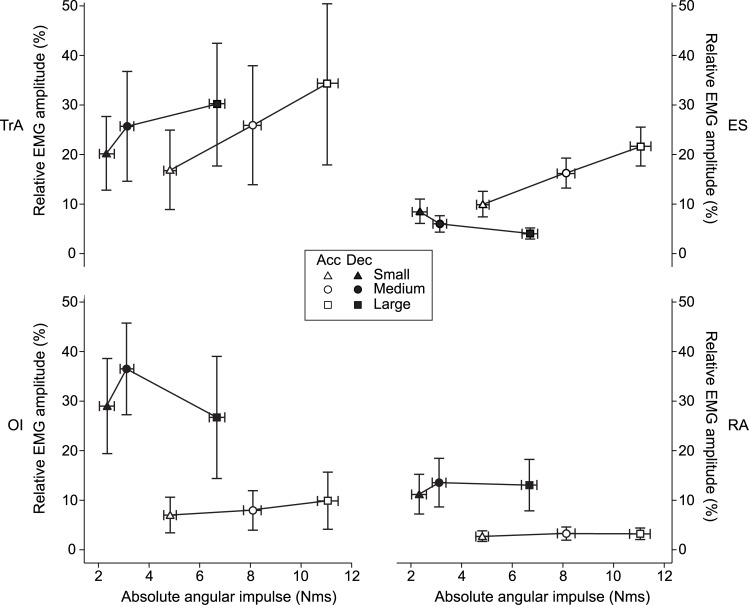
The relationship between EMG amplitude and angular impulse. The relationship between the mean EMG amplitude in % of that at maximum voluntary contraction (95% CI) for the four muscles investigated and the mean absolute angular impulse in Nms (95% CI) for each muscle, arm movement amplitude (small, medium and large) and phase (acceleration and deceleration).

The various trunk muscles displayed different patterns of association between the magnitude of muscle activation and the angular impulse on the trunk (Fig 4). Visual inspection of Fig 4 shows that the activation level of TrA was positively associated with the impulses at the start of both acceleration and deceleration of the arm movement. For ES this was true only at the start of acceleration. For OI and RA across phases, and for ES at the start of deceleration there was no obvious association between the magnitude of activation and the size, or the direction, of the impulses.

## Discussion

The present data showed that trunk muscle behaviour was not constant between different movement amplitudes of rapid bilateral shoulder flexions. By varying the amplitude of rapid shoulder flexion movements in standing, and by considering both the acceleration and the deceleration phases, it was possible to grade the magnitude and direction of perturbation of the trunk induced at shoulder level. This variation in trunk perturbation between different arm movement amplitudes was associated with different modulations in activation patterns among the trunk muscles, both with respect to onset latency and activation magnitude. All abdominal muscles were activated earlier with larger arm movement amplitude, whereas TrA was the only muscle that also showed greater activation magnitude with increased trunk perturbation size, irrespective of perturbation direction.

### Acceleration phase

Increasing the amplitude of a rapid shoulder flexion movement significantly increased the kinematic variables of the arm and the associated reactive forces and torques on the trunk at shoulder level during the acceleration of the arm, initially directed in the flexion direction. This is in concordance with a previous study where the speed of the arm was altered, albeit the amplitude was kept constant, showing greater perturbation with increased speed [15].

The increase in initial perturbation was associated with specific modulations of onset of trunk muscle activation. Although all onset latencies were within the realm of feedforward control, there was a clear shortening (about 20 ms) of abdominal muscle latencies with increased arm movement amplitude from small to large. A “dosage” sensitive muscle response suggests that the activation is dependent on the size of the forthcoming perturbation, indicating a change in preprogramming in anticipation of the larger perturbation. These findings are in concordance with previous studies that show an earlier activation of TrA in association with higher speed of arm movement (movement amplitude unchanged) [15]. However, except for OI, there was no difference between the medium and large amplitude movement in the present study indicating that the modulation of onset latency with movement amplitude reaches a “plateau” where further shortening of the onset latency does not further improve the functional consequences. On the other hand, there was no adaptation in onset latency for ES, which is in line with previous research that showed no variation in onset latency of ES above a certain threshold of arm movement speed during shoulder flexion [25]. Possibly, the “plateau”, mentioned above, is reached earlier for ES than the abdominal muscles, i.e. a modulation in the time domain does not provide any biomechanical advantage.

Interestingly, of the studied abdominal muscles, only TrA showed a modulation of activation magnitude simultaneous to the adaptation in onset latency during the acceleration phase. The increase in TrA activation magnitude for all increments in movement amplitudes and the concomitant increase in ES activation between the small and large movement are compatible with the larger reactive trunk flexion moment induced by the larger arm movements. It is possible that TrA could assist ES in generating an extensor torque on the trunk through an increased intra-abdominal pressure [26] in a synergistic pattern that has been observed before [27], or, that TrA provides a more non-specific contribution to trunk control.

### Deceleration phase

Although kinematic and kinetic information from complete shoulder flexion movements (from start to end) have been presented graphically in several studies [1,2,28], the deceleration of the movement has never been the focus of the experiments. While the acceleration of the arm is performed against the force of gravity, the deceleration of the arm is assisted by gravity to varying degree depending on the stop position, with the greatest assistance in the medium movement in the present study where the forward horizontal displacement of the arms’ COM is the greatest. This might reflect the small change in impulse during the deceleration phase between the small and medium amplitude movement despite higher peak and mean angular deceleration during the medium movement. It can also provide a possible explanation to the lack of increase in activation magnitude for OI and RA with increasing arm movement amplitude. However, the lack of increase, rather a tendency of decreased activation, of these muscles between the medium and large arm movement amplitude when the assistance of gravity decline during deceleration would suggest otherwise. Yet, in the positions where the arms reaches forwards or above the head, particularly the RA but also OI might be stretched so that they are activated in an eccentric manner, which would require less activation levels for the same muscle torque than during concentric work [29].

Another biomechanical prerequisite during the deceleration phase that differs between the different arm movement amplitudes is the postural demand due to the height of the COM over the base of support [28], which is the highest in the large amplitude movement. This aspect is not covered by the inverse dynamics calculations, and represents an increased postural threat, where any perturbing force has a larger destabilizing potential. It has been seen previously in various static arm positions that particularly TrA activation magnitude is closely related to an increase in postural demand [19].

### Acceleration vs. deceleration

Out of the trunk muscles, TrA displayed a unique activation pattern with the same activation magnitude during the deceleration of the arm movement as during the acceleration across movement amplitudes. Hence, it seems that the purpose of TrA activation might vary depending on situation and that it can add to the control of the lumbar spine, not only by contributing to extension (as mentioned above), but also in a direction non-specific manner in the sagittal plane. The independence of the magnitude of TrA activation from the induced direction of a perturbation in the sagittal plane is consistent with previous experiments conducted in standing and side-lying, with static arm positions and sudden external loading to the trunk, respectively [19,30], suggesting that it is not dependent on the task performed or body position.

Besides TrA, all other muscles in the present study were phase specific in their activation with OI and RA showing higher activation levels during deceleration of the arm and ES during acceleration. A likely explanation is the torque generating capacities of these muscles. The fibre orientation of ES makes this muscle suitable to counteract the initial flexion perturbation to the trunk, thus higher activation is required during the acceleration phase than the deceleration phase. Further, the lower activation of ES during the deceleration of the large compared to the small and medium amplitude movement could be influenced by the change in direction of the linear Fx-impulse during the deceleration phase of this movement. During the large movement the mean linear Fx-impulse is posteriorly oriented, which could assist ES and perhaps decrease the need for activation of this muscle. However, following the same reasoning, OI and RA, that generate flexion torque on the trunk, should then increase their activation levels during the deceleration of the large amplitude movement, but this is not supported by the present data. Thus, other aspects of the kinetic chain, not controlled for in this experimental set-up, are likely to contribute to the complex coordination of trunk muscles and further research is needed to gain complete understanding of the biomechanics surrounding the bilateral arm raise.

### Methodological considerations

Since the arm movement paradigm is frequently used to evaluate motor control in low back pain patients, the present study can provide important reference material. However, in this regard it is important to consider that this study included only healthy male subjects, which affects the external validity. Furthermore, we used bilateral arm movements, whereas some previous studies use a unilateral arm flexion. Unilateral arm movements produce a rotational torque to the trunk, which might be responsible for the frequent findings of early activation of the contralateral TrA [31]. However, the fact that larger arm movement amplitude creates a larger perturbation and has an impact on latency and EMG magnitude measurements should be universal.

The choice of placement of the centre of rotation affects the inverse dynamics model. A chosen centre of rotation lower in the trunk, e.g. in the lumbar spine, could perhaps be more intuitive since it is the abdominal and lumbar back muscles that are under study. This would give the linear forces a longer moment arm to the shoulders (the point of application of the linear forces) and a larger impact on the net angular momentum calculations. However, in the present set-up, this would mean that the trunk would have to be reduced to one or two rigid segments, which would be an over-simplification of reality, creating results that would be difficult to interpret.

Intra-muscular EMG is regarded as the most reliable method for obtaining activity recordings from deep muscle layers where there are superficial muscles overlapping, and the results in the present study were statistically robust. Nevertheless, all findings from experimental studies should be interpreted in the light of the measurement error associated with the chosen methodology. Previous results indicate that intra-muscular recordings of TrA and OI mixed together, during single shoulder flexion tasks, has a minimal detectable change (SDD) in latency of 29 ms (i.e. two single repetitions lies within this interval with 95% certainty) [32]. However, it is unclear if the arm movement amplitude in that study was standardized, which, if not, in combination with the small subset of participants (n = 5) indicates (as the authors point out) that variability might be overestimated [32]. This is supported with other findings from the same study, where the average latency difference between two sets of shoulder flexions (separated by 30 min) was only 1 ms (95% CI of bias; ±12 ms) [32]. In conjunction with these considerations, the mean of three repetitions was used in the calculations in the present study, and not single contractions, which further suggests that the indicated SDD is too conservative to be used in the present setting. Therefore we believe that the current statistically significant findings of latency differences between arm movement amplitudes exceed what can be expected to be a measurement error.

### Practical implications

The present results show that movement amplitude needs to be standardized when performing rapid shoulder flexions to evaluate trunk muscle activation. This needs to be considered regardless of whether the focus lies on the activation of the trunk muscles in the initial or later phases of the movement, on within subject designs, on comparing activation of the trunk muscles between categories of subjects, or, above all on trying to synthesize findings from different studies. In addition, inclusion of the deceleration of the arm movement in the analysis can provide an opportunity to study the relationship between trunk muscle activation and direction of perturbing torque during one and the same arm movement.

### Conclusion

Increased movement amplitude of rapid bilateral shoulder flexion affects initial trunk muscle responses with shortened onset latencies of the abdominal muscles and increased level of activation in TrA and ES. Shoulder flexion movements impose a flexion angular impulse to the trunk at shoulder height during acceleration of the arms, which is reversed during deceleration. Despite this change in trunk perturbation direction, TrA activation remains on the same level, whereas the other trunk muscles show a response specific to either acceleration or deceleration. This lends support to the notion that TrA is involved in the control of the spine in a way not necessarily related to the direction of the perturbation in the sagittal plane.
